# Roscovitine Suppresses CD4+ T Cells and T Cell-Mediated Experimental Uveitis

**DOI:** 10.1371/journal.pone.0081154

**Published:** 2013-11-18

**Authors:** Zili Zhang, Qi Liu, Konstantin S. Leskov, Xiumei Wu, Jie Duan, Gary L. Zhang, Mark Hall, James T. Rosenbaum

**Affiliations:** 1 Department of Pediatrics, Case Western Reserve University, Cleveland, Ohio, United States of America; 2 Department of Pediatrics, Oregon Health & Science University, Portland, Oregon, United States of America; 3 Departments of Medicine and Ophthalmology, Oregon Health & Science University, Portland, Oregon, United States of America; 4 Devers Eye Institute, Legacy Health System, Portland, Oregon, United States of America; National Eye Institute, United States of America

## Abstract

**Background:**

T cells are essential for the development of uveitis and other autoimmune diseases. After initial activation, CD4+ lymphocytes express the co-stimulatory molecule OX40 that plays an important role in T cell proliferation. Cyclin dependent kinase 2 (CdK2) plays a pivotal role in the cell cycle transition from G1 to S phase. In addition, recent research has implicated CdK2 in T cell activation. Thus, we sought to test the immunosuppressive effect of roscovitine, a potent CdK2 inhibitor, on CD4+ T cell activation, proliferation, and function.

**Design and Methods:**

Mouse CD4+ T cells were activated by anti-CD3 and anti-CD28 antibodies. The expression of OX40, CD44, and CdK2 were analyzed by flow cytometry. In addition, cell cycle progression and apoptosis of control and roscovitine-treated T lymphocytes were measured by BrdU incorporation and annexin V assay, respectively. Furthermore, the immunoregulatory effect of roscovitine was evaluated in both ovalbumin-induced uveitis and experimental autoimmune uveitis (EAU) models.

**Results:**

In this study, we found that T cell activation induced OX40 expression. Cell cycle analysis showed that more CD4+OX40+ cells entered S phase than OX40- T cells. Concurrently, CD4+OX40+ cells had a higher level of CdK2 expression. Roscovitine treatment blocked activated CD4+ cells from entering S phase. In addition, roscovitine not only reduced the viability of CD4+ lymphocytes but also suppressed T cell activation and cytokine production. Finally, roscovitine significantly attenuated the severity of T cell-dependent, OX40-enhanced uveitis.

**Conclusion:**

These results implicate CdK2 in OX40-augmented T cell response and expansion. Furthermore, this study suggests that roscovitine is a novel, promising, therapeutic agent for treating T cell-mediated diseases such as uveitis.

## Introduction

T lymphocytes play an important role in the pathogenesis of many autoimmune diseases including uveitis by recognizing antigens and orchestrating the immune response. Upon encountering antigens, activated naïve T cells differentiate into effector lymphocytes. This differentiation process is usually coupled with the clonal expansion of responding T cells, a critical step for the exponential increase of activated lymphocyte number to meet the immunological demand.

At the time of activation, T cells express an array of co-stimulatory molecules, and the engagement of these co-stimulatory molecules not only elicits the T cell response but also facilitates clonal expansion. For instance, OX40 (CD134), a co-stimulatory molecule in the TNF receptor superfamily, is expressed by activated T cells. In addition to enhancing T cell effector function, OX40 promotes cell proliferation and survival, leading to the expansion of lymphocyte populations. OX40 signals through the phosphoinositide 3-kinase (PI3K)-AKT-mTOR pathway [1-3]. In addition, it is postulated that OX40 co-stimulation enhances the expression or function of cyclins and cyclin-dependent kinases (CdKs) [4]. However, currently there is no published study showing the up-regulation of CdKs in OX40+ lymphocytes. OX40 has been used as a marker for T cell activation.

CdKs are a group of serine/threonine kinases pivotal for controlling cell cycle and mitosis. When quiescent cells enter the G1/S phase, the synthesis of cyclins D and E is temporarily increased. Cyclin D interacts with CdK4 and CdK6 to drive the cells from G_0_ through mid-G_1_ phase [5,6]. In contrast, CdK2, also known as cell division protein kinase 2, is primarily expressed during the mid and late-G_1_ phase [7]. CdK2 binds Cyclin E and plays an important role in G1 to S transition, while its interaction with Cyclin A facilitates the cells to progress through the S phase [8,9]. Because of their indispensible role in the cell division, CdKs are essential for T cell clonal expansion [10]. It has been shown that CdK4 and CdK6 inhibitor blocks αβ T cell proliferation and differentiation [11]. However, the involvement of CdK2 in lymphocyte expansion has not been extensively studied. Rowell et al. reported that the genetic deletion of the CdK2 endogenous inhibitor, p27(Kip1), results in the loss of T cell immune tolerance [12]. Furthermore, a recent study suggests that inhibition of CdK2 leads to diminished IL-2 and IFN-γ production in CD4+ T cells and enhancement of allograft survival [13]. These findings indicate that CdK2 regulates not only lymphocyte proliferation but also T cell activation and function. 

Roscovitine is an antiproliferative agent. It functions as a purine analog to interfere with ATP binding to CdKs. Roscovinte exhibits a potent inhibitory effect on CdK2 activity, and was originally designed for suppressing tumor cell growth and division [14]. However, several recent studies have shown that roscovitine down-regulates effector immune cells such as eosinophils and neutrophils, thereby reducing inflammation [15-17]. Nevertheless, the therapeutic effect of roscovitine on T lymphocytes has not been well defined. Therefore, the purpose of this study is to evaluate the impact of roscovitine on the proliferation, survival, and function of activated CD4+ T cells. First, we defined T cell activation by measuring the expression of OX40 and CD44. Next, we showed that CD4+OX40+ T cells displayed a higher proliferation rate and CdK2 level than OX40- cells. Roscovitine treatment arrested the cell cycle progression of CD4+ lymphocytes. Furthermore, the CdK inhibitor enhanced apoptosis and inhibited activation and cytokine production by CD4+ T cells. Lastly, we augmented T cell activation and proliferation in mouse uveitis models using OX40 activating antibody. Roscovitine significantly attenuated ocular inflammation in the setting of T cell-mediated uveitis. Taken together, these findings suggest an inhibitory effect of roscovitine that correlates with a reduction in CdK2 in activated/proliferating CD4+ T cells. This study also suggests the therapeutic potential of roscovitine for treating autoimmune uveitis and other T cell-mediated diseases. 

## Materials and Methods

### Animals

Six to 8 week-old C57BL/6, BALB/c and DO11.10 mice on a BALB/c background (Jackson Laboratory, Bar Harbor, Maine) were housed at Case Western Reserve University and Oregon Health & Science University, respectively. The animal experimental protocol has been approved by the institutional animal care and use committee of Case Western Reserve University and Oregon Health & Science University (IACUC numbers 2012-0178 and ISO1307). 

### Cell Culture, Isolation, and Stimulation

After C57BL/6 and DO11.10 mice were sacrificed, their spleens were removed. Single cell suspensions were prepared by passing the tissue through a 70 μm cell strainer (BD Biosciences, Mountain View, CA). The cell suspension was washed twice with RPMI 1640, and then cultured in RPMI 1640 with 10% fetal bovine serum (FBS) in an atmosphere of 95% air and 5% CO_2_ at 37°C.

To activate the T cells from C57BL/6 mice, cell culture plates were coated with 5μg/ml anti-mouse CD3 antibody (eBioscience, San Diego, CA) at 4°C over night. Then, 2 x 10^6^/ml C57BL/6 splenocytes were incubated in the anti-CD3 antibody-bound plates along with 2 μg/ml anti-mouse CD28 antibody (eBioscience) in the culture media. 

DO11.10 splenocytes were activated with 5 μg/ml OVA_323-339_ peptide (Anaspec, San Jose, CA), and some cells were further stimulated with OX40 activating antibody (Clone OX86). The antibody was kindly provided by Dr. Andrew Weinberg from the Earle A. Chiles Research Institute at Providence Cancer Center. This monoclonal antibody is a rat IgG1 that specifically interacts with mouse OX40, leading to the enhancement of T cell activation and function [18]. Furthermore, this antibody promotes a T cell response in wild-type mice but not in OX40 knockout animals, suggesting that this agonistic antibody specifically activates OX40 [18]. For adoptive transfer, these lymphocytes were further purified using Lympholyte®-M (Cedar Lane Laboratories, Burlington, NC) according to the manufacturer’s instructions. After the purification, 85-90% of DO11.10 lymphocytes were CD4+ T cells.

### Flow Cytometry

C57BL/6 splenocytes were suspended in PBS containing 2% FBS and 0.1% sodium azide. Fluorescein isothiocyanate (FITC)-conjugated anti-mouse CD4 (Clone GK1.5), phycoerythrin (PE)-conjugated anti-mouse CD44 (Clone IM7), and allophycocyanin (APC)-conjugated anti-mouse OX40 (Clone OX86) antibodies (eBioscience, San Diego, CA) were used to label these cell surface markers according to the manufacturer’s instruction. 

### Cell Cycle Analysis

The splenocytes were incorporated with 5-bromo-2'-deoxyuridine (BrdU) for 1 hour to label newly synthesized DNA (BD Bioscience). Then, the cells were fixed and stained with fluorescent-conjugated anti-BrdU antibody and 7-amino-actinomycin D (7-AAD), a dye that binds to total DNA. Incorporated BrdU and total DNA levels were determined by flow cytometry to quantify the CD4+ lymphocytes in different cell cycle phases in accordance with the manufacturer's instructions (BD Biosciences). 

### Annexin V and Propidium Iodide Staining

Cell apoptosis and death were detected by double staining for annexin V and propidium iodide (PI) (eBioscience). After washing with PBS, lymphocytes were suspended in binding buffer (10 mM Hepes/NaOH, pH 7.4, 140 mM NaCl, 2.5 mM CaCl,). The cells were labeled with FITC-annexin V and PI for 15 min at room temperature in the dark according to the manufacturer’s instruction, and the samples were analyzed by flow cytometry.

### Western Blotting

To extract nuclear Poly (ADP-ribose) Polymerase (PARP), 1 x 10^6^ activated lymphocytes were lysed in extraction buffer (62.5 mM Tris-Cl, pH 6.8, 4M Urea, 10% Glycerol, 2% SDS, 0.00125%, bromophenol blue, and 5% β-mercaptoethanol). For detecting cytoplasmic proteins, the cells were collected in 1X LDS lysis buffer (Invitrogen, Carlsbad, CA) on ice. The lysates were then centrifuged at 12,000 g for 10 min. The lysates were then heated and centrifuged at 12,000g for 10 min. Thirty microliters of total protein from each group were separated by electrophoresis through a 4–12% gradient Tris–glycine SDS gel, and then transferred to nitrocellulose membrane. After milk blocking, the nitrocellulose membrane was incubated with the polyclonal antibody against PARP (Cell Signaling Technology, Danvers, MA), the growth arrest and DNA damage-inducible protein 45- β (Gadd45β) or β-actin (Santa Cruz Biotechnology, Santa Cruz, CA), followed by HRP-conjugated secondary antibody. The signals of PARP, Gadd45β, and β-actin were detected by enhanced chemiluminescence luminol reagent.

### ELISA

Fifty µl culture media of cultured lymphocytes from various experimental groups were collected for ELISA to measure IFN-γ- and IL-17 production according to the manufacturer’s protocols (BioLegend, San Diego, CA).

### Induction of OVA Uveitis

For the adoptive transfer model of uveitis, OVA-activated DO11.10 lymphocytes with and without OX40 activating antibody priming were injected into naive BALB/c mice (1.5 × 10^7^ cells/animal) via the tail vein. Then, the recipient mice were challenged intravitreally with 100 µg of OVA (Sigma-Aldrich, St. Louis, MO). Forty eight hours after the OVA challenge, uveitis was evaluated by intravital microscopy.

### Intravital Microscopy

As previously described [19,20], 150 μl of rhodamine (0.2% in PBS) was administered intraperitoneally into adoptive transfer recipient mice to label intravascular leukocytes immediately before intravital microscopy. Labeled inflammatory cells in the iris and ciliary/limbal region were observed by intravital epifluorescence videomicroscopy. This imaging system was comprised of a modified DM-LFS microscope (Leica, Bannockburn, IL) and a CF 84/NIR B&W camera from Kappa (Gleichen, Germany). Real-time videos were recorded in NTSC format for 10 seconds each. Both rolling and adherent leukocytes in the iris vessels were identified as a marker for anterior chamber uveitis [19,20]. These cells were quantified to assess the severity of the ocular inflammation [19,20].

### Induction of Experimental Autoimmune Uveitis

For EAU, B10.RIII mice received subcutaneous immunization (near the base of the tail) of 50 µg interphotoreceptor retinoid-binding protein (IRBP)_161-180_ peptide (Ser-Gly-Ile-Pro-Tyr-Ile-Ile-Ser-Tyr-Leu-His-Pro-Gly-Asn-Thr-Ile-Leu-His-Val-Asp) (AnaSpec) in 200 µl complete Freund’s adjuvant (Sigma-Aldrich) with *Mycobacterium tuberculosis* strain H37RA. On Day 21, the eyes were harvested and the severity of EAU was examined by histology, and graded on a four-point scale based on inflammatory cell infiltration, retinal folding and destruction [21].

### Histology

On day 21 after EAU induction, the mice were euthanized. Enucleated eyes were fixed in 3% paraformaldehyde. Then, the tissues were embedded in paraffin, sectioned, and stained with haematoxylin and eosin (H&E). Retinal inflammation was assessed by light microscopy.

### Statistics

Data are expressed as the average ± SD, and a representative experiment is shown for each figure. Statistical probabilities were evaluated by Student’s *t* test or ANOVA, with a value of *p* < 0.05 considered significant. For EAU scoring, median difference between control and experimental groups was compared using the Mann-Whitney U test.

## Results

### Activated CD4+ T Cells Express OX40 and CdK2 and Are in an Active Proliferation State

**Figure 1:**
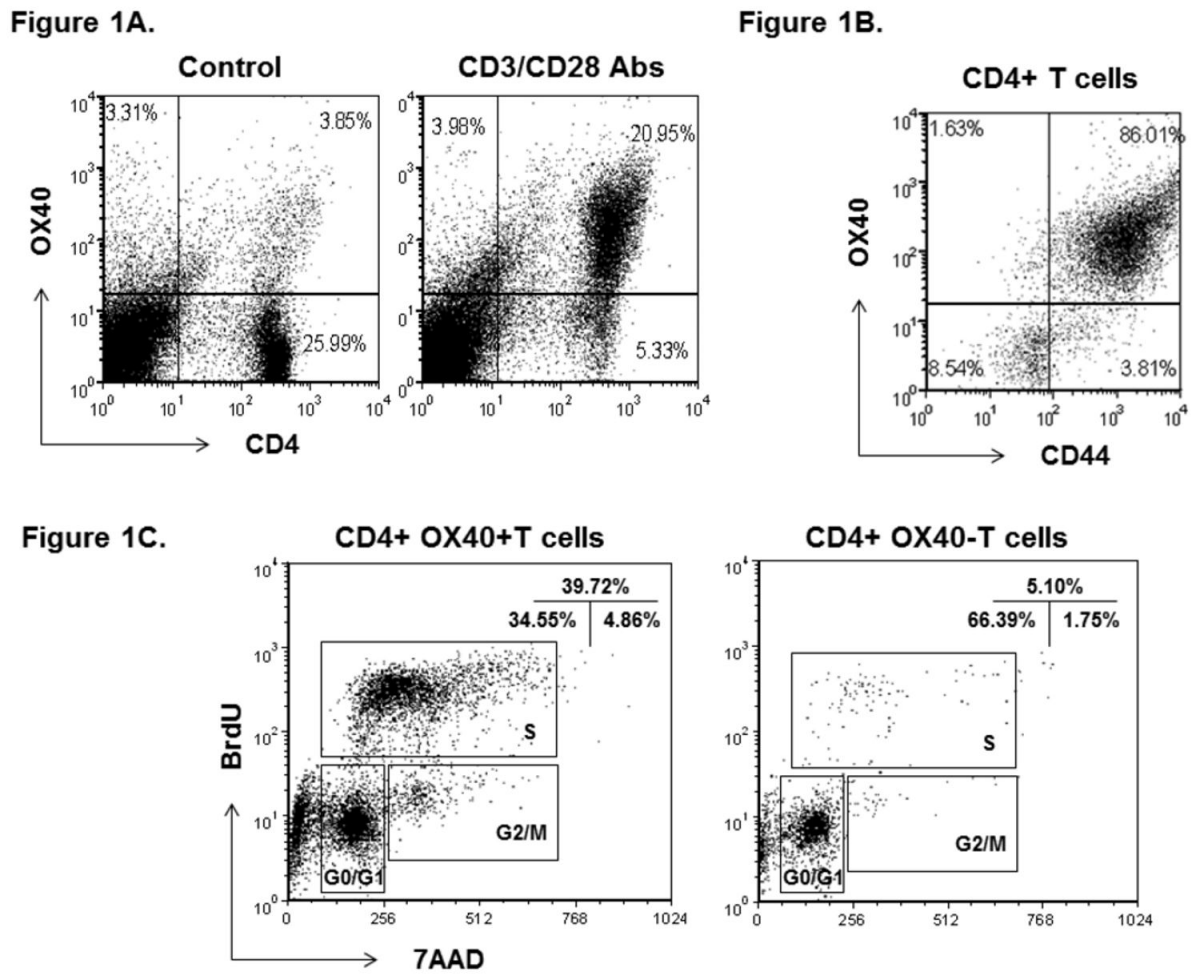
Expression of OX40 in activated CD4+ T cells (A). Splenocytes from C57BL/6 mice were activated with anti-CD3 and anti-CD28 antibodies *in* *vitro* for 72 hours. CD4 and OX40 were analyzed by flow cytometry. Shown are representative plots of OX40 level in gated CD4+ lymphocytes from 3 independent studies. Expression of CD44 in CD4+OX40+ cells (B). Surface expression of CD44 of C57BL/6 splenocytes was examined at 72 hours after anti-CD3 and anti-CD28 antibody treatment. Shown are representative plots of CD44 and OX40+ expression in gated CD4+ lymphocytes from 3 independent studies. Cell cycle analysis of CD4+OX40+ and CD4+OX40- lymphocytes (C). After 72-hours of anti-CD3 and anti-CD28 antibody stimulation, activated lymphocytes were first stained for surface CD4 and OX40, followed by BrdU and 7-AAD labeling. Different cell cycle phases were analyzed by flow cytometry and compared between OX40+ and OX40- T cells. Shown are representative plots of cell cycle analysis of gated CD4+OX40+ and CD4+OX40- lymphocytes from 3 independent studies.

**Figure 2:**
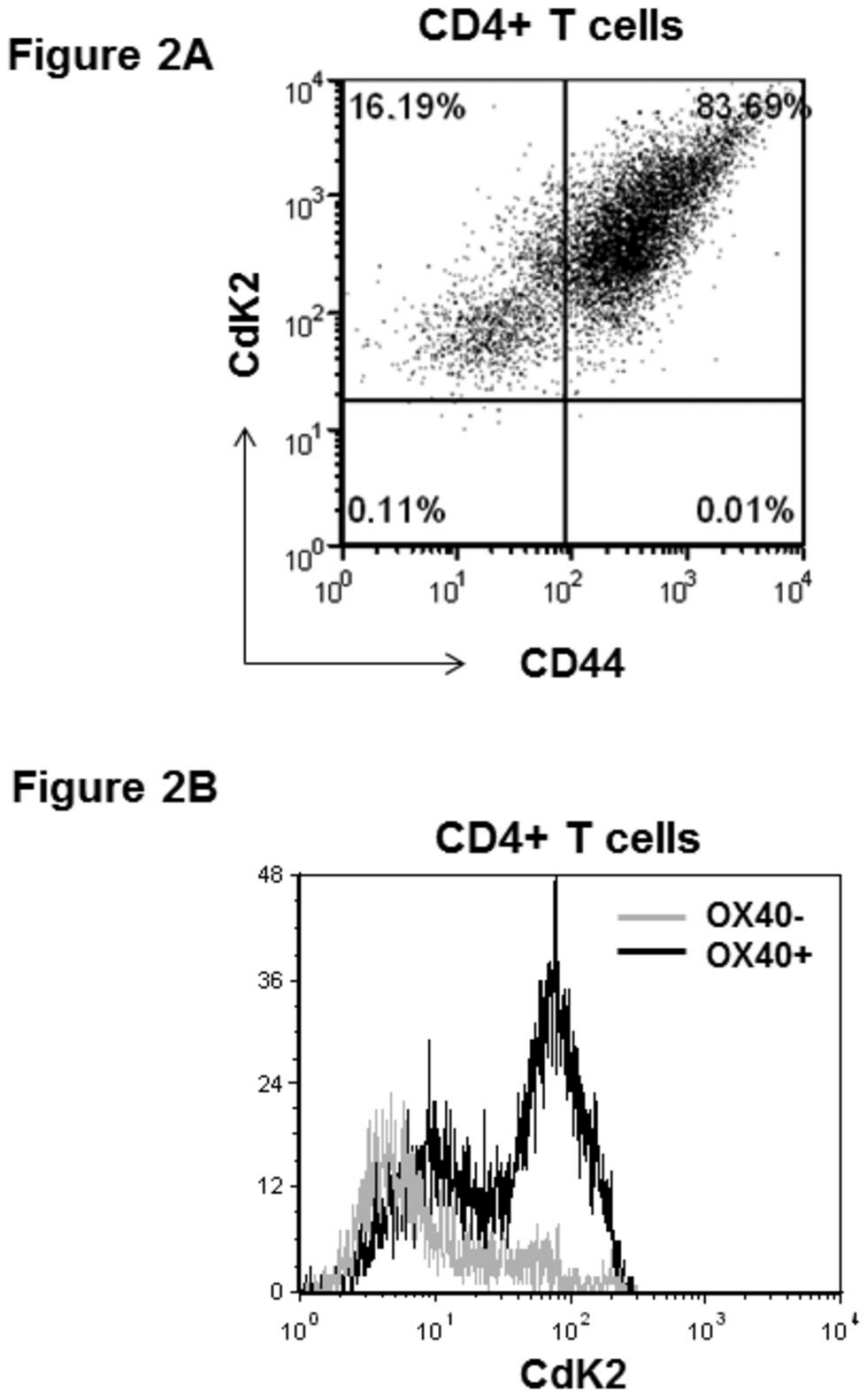
Expression of CdK2 in activated CD4+CD44+ cells (A). C57BL/6 splenocytes were activated with anti-CD3 and anti-CD28 antibodies *in* *vitro*. Seventy-two hours later, surface CD4, CD44 and intracellular CdK2 were stained and analyzed by flow cytometry. Shown are representative plots of CdK2 and CD44 expression in gated CD4+ lymphocytes from 3 independent studies. Higher CdK2 expression in CD4+OX40+ T cells (B). Splenocytes from C57BL/6 mice were activated with anti-CD3 and anti-CD28 antibodies *in* *vitro* for 72 hours. Cell surface CD4 and OX40 expression were analyzed by flow cytometry. Gated CD4+OX40- and CD4+OX40+ cells were further analyzed for the intracellular expression of CdK2. Shown are representative plots from 3 independent studies.

 Previous studies demonstrated that T cell activation induces OX40 expression [22]. In order to evaluate the effect of roscovitine on T cell activation, we first identified activated T cells by measuring the expression of OX40 as an activation marker. Splenocytes of C57BL/6 mice were stimulated with anti-CD3 antibody (5 µg/ml) and anti-CD28 antibody (2 µg/ml). Seventy-two hours later, surface OX40 on CD4+ lymphocytes was analyzed by flow cytometry. During resting state, a few CD4+ T cells expressed OX40, whereas the induction of OX40 was up-regulated in approximately 80% of CD4+ cells after anti-CD3 and anti-CD28 antibody activation (Figure 1A). To further characterize the activation state of CD4+OX40+ cells, we measured the expression of the activation marker CD44 in these lymphocytes. As shown in Figure 1B, a majority of CD4+OX40+ cells were positive for CD44 staining, suggesting that OX40+ lymphocytes are an activated population. Since OX40 is implicated in lymphocyte proliferation, we went further to assess if these activated T cells displayed a higher state of proliferation. After 72-hour anti-CD3 and anti-CD28 antibody activation, we used BrdU incorporation to analyze cell cycle progression in both CD4+OX40+ and OX40- T cells. Compared to CD4+OX40- lymphocytes, OX40+ cells had an approximately 8-fold increase of its population in S phase, suggesting that OX40-expressing T cells are more proliferative (Figure 1C). In light of this finding, we then asked if activated CD4+ lymphocytes express CdK2. We chose CdK2 because it has recently been implicated in T cell function [9,12,23]. In addition, CdK2 is a pivotal regulator promoting lymphocytes from G1 phase to S phase [8]. First, the intracellular staining of CdK2 was measured by flow cytometry. As illustrated in Figure 2A, more than 80% of CD4+CD44+ lymphocytes were positive for CD44 and intracellular CdK2 staining. Furthermore, we compared the intracellular level of CdK2 between CD4+OX40+ and CD4+OX40- lymphocytes. As shown in Figure 2B, flow cytometry analysis revealed that CD4+OX40+ T cells expressed a higher level of CdK2 than the OX40- counterpart. These data indicate that activation of T cells as measured by OX40 and CD44 induction is associated with cell cycle progression, and it further coincides with the expression of CdK2. Although OX40 is presumably linked to CdK activation, this is the first known study showing that OX40+ lymphocytes express a higher level of CdK2.

### Roscovitine Inhibits the Cell Cycle Progression of Activated CD4+ T Cells

**Figure 3:**
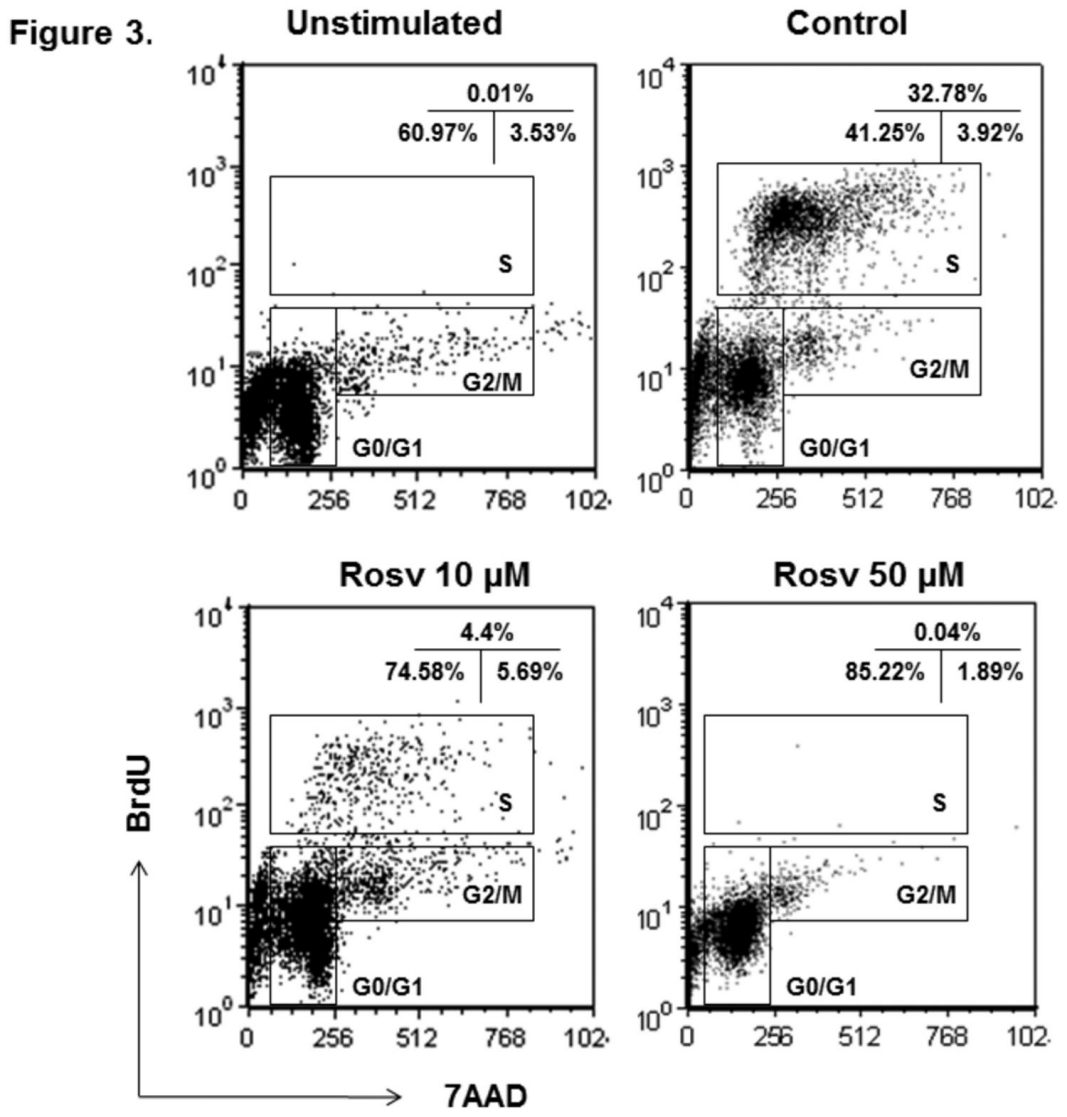
Blocking of activated CD4+ T lymphocyte cell cycle progression by roscovitine. Splenocytes from C57BL/6 mice were activated with anti-CD3 and anti-CD28 antibodies *in* *vitro* in the presence of 0, 10, or 50 μM roscovitine. Then, BrdU incorporation and cell cycle analysis were performed at 72 hours after T cell activation. Shown are representative plots of cell cycle analysis of gated CD4+ lymphocytes from 4 independent studies.

 In light of above findings, we postulated that CdK2 drives the transition from G0/1 to S phase when activated lymphocytes enter expansion phase. In addition, it is feasible to investigate if inhibition of CdK2 down-regulates T cell response. Thus, we first sought to determine whether CdK2 inhibitor roscovitine suppressed cell cycle progression of activated CD4+ lymphocytes. Figure 3 shows the flow cytometry analysis of BrdU incorporation and cell cycles of CD4+ T cells. Compared to unstimulated lymphocytes, a significant portion of activated CD4+ cells entered S phase after anti-CD3 and anti-CD28 antibody treatment (Figure 3). However, roscovitine substantially reduced the cell number in S phase. Concomitantly, more activated T cells accumulated in G1 phase (Figure 3). This result suggests that roscovitine suppresses CD4+ lymphocyte proliferation by mainly blocking G1 to S cell cycle transition. 

### Roscovitine Induces CD4+ T Cell Death in a Time- and Dose-dependent Manner

**Figure 4:**
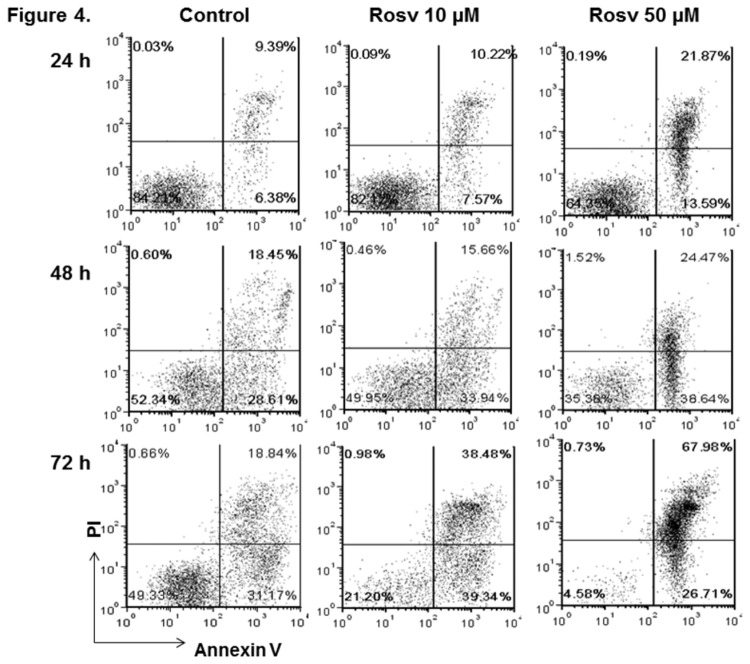
Induction of CD4+ T lymphocyte apoptosis and cell death by roscovitine. Splenocytes from C57BL/6 mice were activated with anti-CD3 and anti-CD28 antibodies *in* *vitro* in the presence of 0, 10, and 50 μM roscovitine. Then, the cells were labeled with annexin V and PI at 24, 48, and 72 hours. Shown are representative plots of a cell viability assay on gated CD4+ lymphocytes from 3 independent studies.

 As discussed above, we have shown that roscovitine blocked cell cycle progression during the activation of CD4+ lymphocytes. Recent studies have demonstrated that roscovitine treatment can also cause apoptosis of B cells, eosinophils, and neutrophils [15-17]. Therefore, we asked if roscovitine could affect the viability of CD4+ T cells. The splenic lymphocytes from C57BL/6 mice were activated with anti-CD3 and anti-CD28 antibodies as described previously, and further cultured in the presence of the 0, 10, and 50 µM roscovitine. To determine cell viability, CD4+ T cells were labeled with annexin V and PI and subsequently analyzed by flow cytometry at 24, 48, and 72 hours. As shown in Figure 4, the number of early apoptotic (annexin V+/PI-) cells and late apoptotic/necrotic (annexin V+/PI+) cells in CD4+ population increased over the time of anti-CD3 and anti-CD28 antibody activation, and activation-induced cell death is mainly responsible for the CD4+ cell death in the control group [24,25]. However, roscovitine treatment significantly increased the annexin V+/PI- and annexin V+/PI+ fractions of activated T lymphocytes. The rise of apoptotic and necrotic CD4+ cells was influenced by the time and dose of roscovitine treatment (Figure 4). 

**Figure 5:**
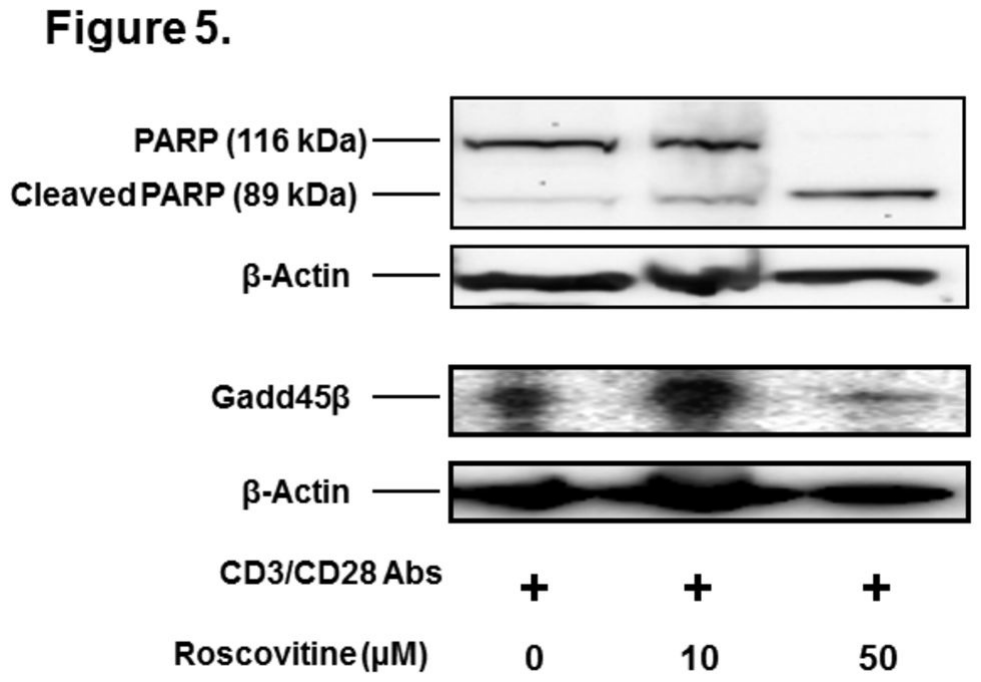
Western blot analysis of cleaved PARP in the lymphocytes treated with roscovitine. Mouse CD4+ T cells were stimulated with anti-CD3 and anti-CD28 antibodies in the presence of 0, 10, and 50 μM roscovitine. Seventy two hours later, PARP and Gadd45β in the cell lysates were analyzed by western blot. In addition, β-actin was measured as a loading control. Shown is the representative image of western blot analysis from 2 independent studies.

PARP is a key enzyme involved in DNA repair and programmed cell death. When the cells undergo apoptosis, PARP (116 kda) will be processed by activated caspsase to cleaved form (89 kDa). The proteolytic cleavage of PARP has been widely used as a marker of apoptosis [26,27]. Recent studies show that roscovitine induces cell apoptosis via a caspase-dependent mechanism [15-17]. Thus, we sought to determine if roscovitine treatment would enhance the cleavage of PARP in the splenic lymphocytes stimulated with anti-CD3 and anti-CD28 antibodies. As illustrated in Figure 5, a trace band of cleaved PARP was observed in the control cells. This is likely due to the apoptosis of some lymphocytes after activation. However in line with the flow cytometry result, 72-hour treatment with roscovitine resulted in a substantial increase of cleaved PARP. The proteolytic conversion of full-length PARP to the apoptotic fragment by roscovitine is also concentration-dependent (Figure 5). Taken together, our study has demonstrated that roscovitine causes not only cell cycle arrest but also cell apoptosis/death of CD4+ lymphocytes.

Gadd45β is a member of Gadd45 family, which is a unique group of stress sensor proteins. They play an important role in cell cycle arrest, DNA repair, cell survival and apoptosis [28]. Thus, we sought to study if roscovitine treatment alters the production of Gadd45β. Compared to control group, 10 µM roscovitine enhanced Gadd45β level in the activated lymphocytes, which may reflect the cellular stress in response to the CdK inhibitor (Figure 5). In contrast, 50 µM roscovitine markedly suppressed the expression of Gadd45β (Figure 5). The attenuation of Gadd45β coincides with the significant increase of lymphocyte apoptosis and death by roscovitine at this dosage (Figure 4). 

### Roscovitine Inhibits T Cell Activation and Response

**Figure 6:**
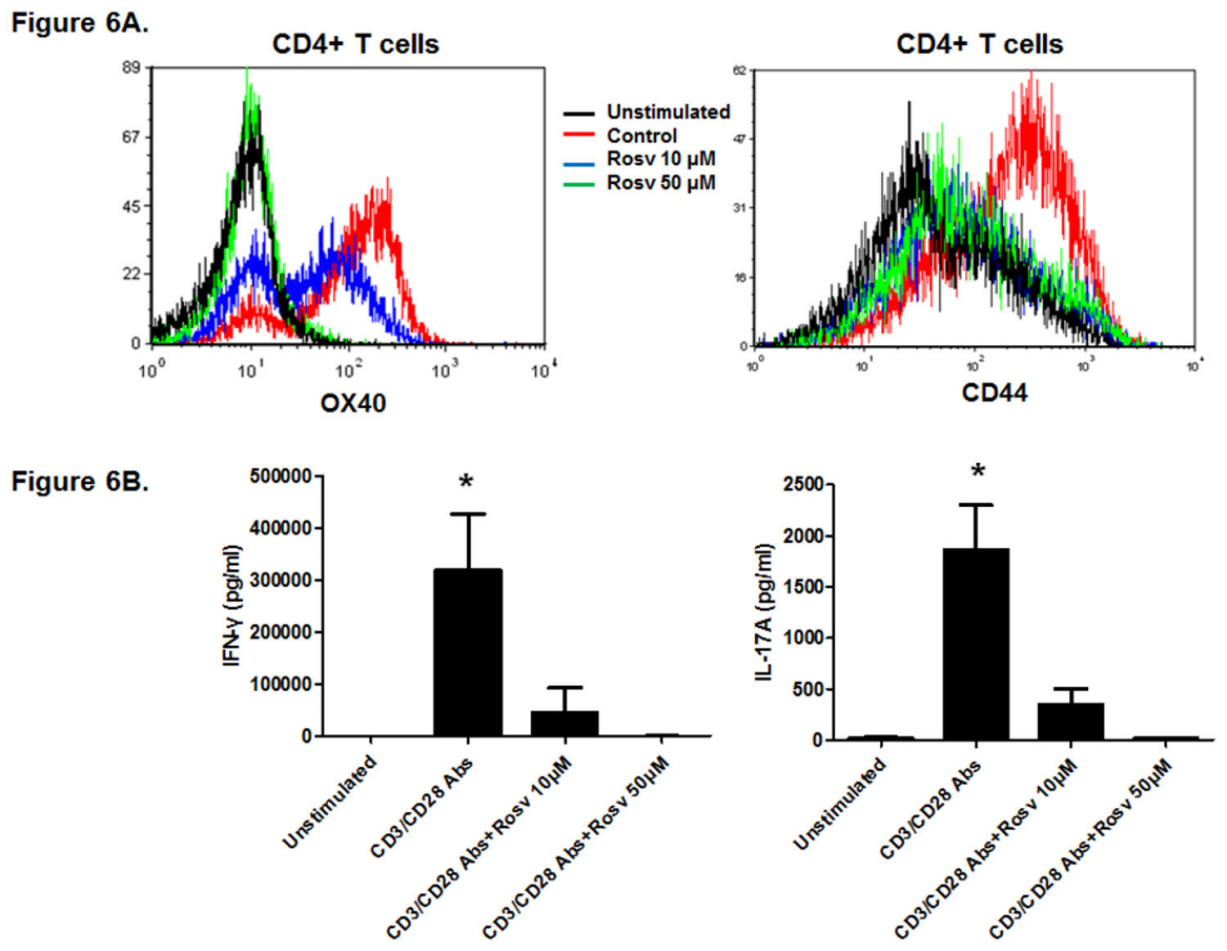
Inhibition of CD4+ T cell activation by roscovitine (A). Splenocytes from C57BL/6 mice were activated with anti-CD3 and anti-CD28 antibodies *in* *vitro*. In addition, the cells were also treated with 0, 10, or 50 μM roscovitine. Seventy-two hours later, surface expression of OX40 and CD44 was examined by flow cytometry. Shown is a representative plot of gated CD4+ lymphocytes from 2 independent studies. Inhibition of IFN-γ- and IL-17 production in activated lymphocytes by roscovitine. The lymphocytes were activated and treated with roscovitine for 72 hours as described previously. Then, secreted IFN-γ- and IL-17 in the culture media were measured by ELISA. The graphs represent average data from 4 mice per group (* p < 0.05).

 Next, we investigated the impact of roscovitine on T cell activation and response *in vitro*. C57BL/6 splenocytes were stimulated with anti-CD3 and anti-CD28 antibodies in the presence and absence of roscovitine for 72 hours. Then, the expression of OX40 and CD44 on CD4+ cells was measured by flow cytometry. As shown in Fig. 6A, T cell activation markedly induced OX40 and CD44 expression in CD4+ cells. However, roscovitine significantly attenuated the induction of OX40 and CD44 in CD4+ T cells, and roscovitine-treated cells had a minimal expression of these T cell activation markers compared to unstimulated lymphocytes (Figure 6A). This suggests that roscovitine suppresses the activation of CD4+ T cells. 

Recent studies have demonstrated that Th1 and Th17 cells both play an important role in the pathogenesis of autoimmune uveitis [29]. Thus, we sought to determine if roscovitine treatment affects the production of Th1 and Th17 cytokines. C57BL/6 lymphocytes were stimulated with anti-CD3 and anit-CD28 antibodies in the presence and absence of roscovitine for 72 hours. Then, IFN-γ (Th1) and IL-17 (Th17) in the culture supernatants were measured by ELISA. No baseline release of IFN-γ or IL-17 was detected in unstimulated lymphocyte culture (Figure 6B). Compared to the unstimulated group, anti-CD3 and anti-CD28 activation promoted IFN-γ and IL-17 production, which was substantially suppressed by roscovitine. The cells treated with 50 µM roscovitine failed to produce IFN-γ and IL-17 at a biologically significant level (Figure 6B). These data suggest that roscovitine inhibits T cell activation and response in addition to blocking their proliferation.

### Roscovitine Attenuates Uveitis Mediated by Activated T cells

**Figure 7:**
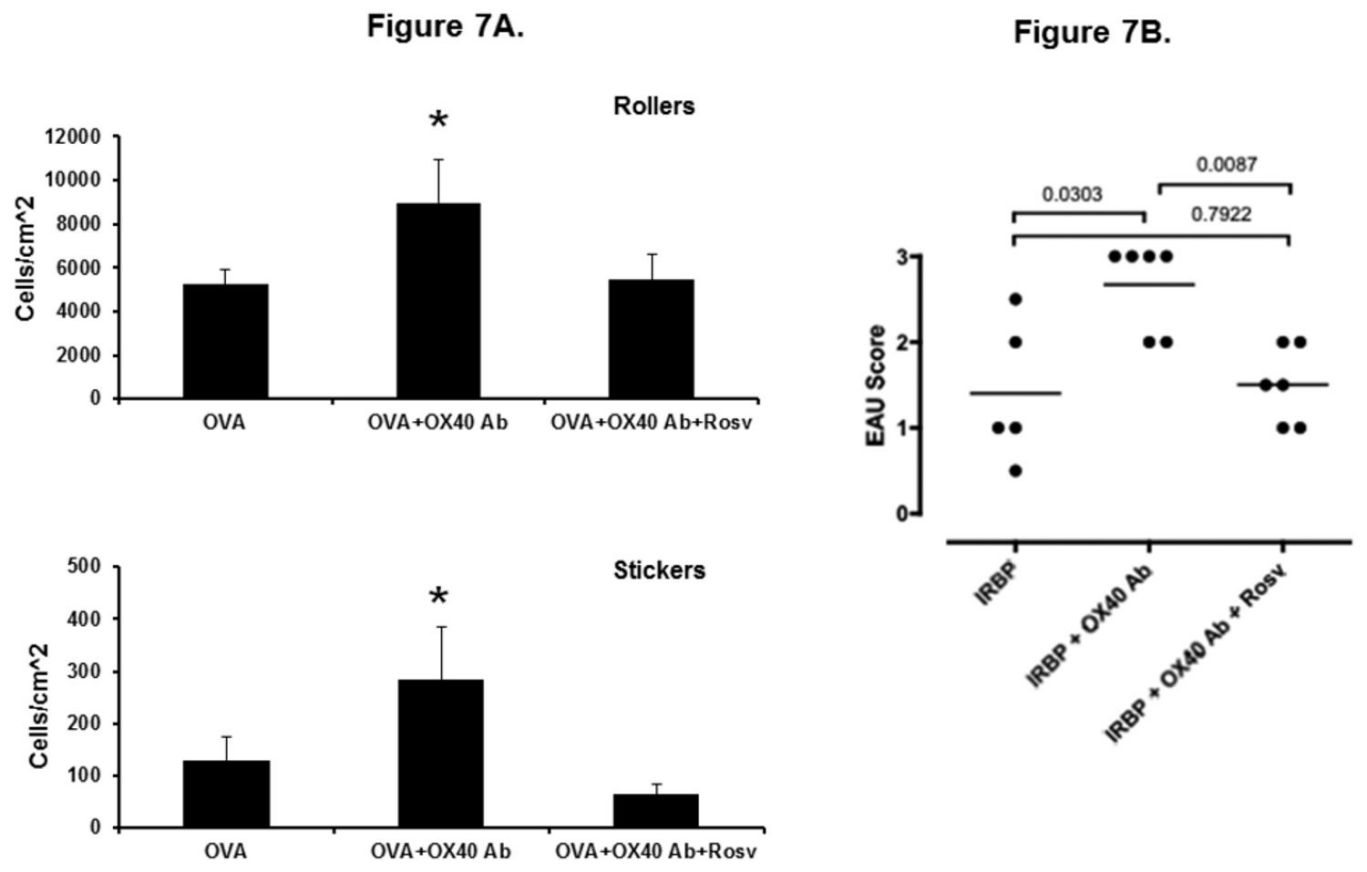
Attenuation of OVA-induced uveitis by roscovitine (A). DO11.10 splenocytes were activated with 4 μg/ml OVA_323-339_ peptide *in* *vitro* in the presence and absence of 4 μg/ml OX40 activating antibody for 3 days. In addition, some cells were treated with 25 μM roscovitine *in* *vitro*. Then, these cells were transferred to BALB/c mice through tail vein injection, followed by intravitreal administration of 100 μg OVA into the eye of recipient mice (n = 5-8 mice per group). Forty eight hours later, ocular inflammatory cells were assessed by intravital microscopy. Both rolling and adherent (sticking) cells in the vasculature of the iris were quantified (* p < 0.05). Amelioration of EAU by roscovitine (B). EAU was induced in B10.RIII mice by subcutaneous injection of IRBP_161-180_ peptide in complete Freund’s adjuvant with *Mycobacterium tuberculosis*. Roscovitine (50 µg per mouse) was administered intraperitoneally every other day from days 0 to 21 after IRBP immunization. On Day 21, the mice were euthanized. Eyes were harvested for histological EAU scoring (Each dot represents left eye from each mouse in different experimental groups).

Next, we examined the *in vivo* effect of roscovitine on T cell function using an adoptive transfer uveitis model developed in our laboratory [22]. OVA_323-339_ specific T cells were harvested from the spleen of DO11.10 mice, and stimulated *in vitro* with the OVA peptide in the presence or absence of OX40 activating antibody for 72 hours. The purpose of priming the donor lymphocytes with OX40 activating antibody is to further enhance their activation and effector function [22]. To validate the *immunomodulatory* effect of roscovitine on T cells, some DO11.10 lymphocytes were treated with roscovitine during the 72-hour stimulation with OVA and OX40. Then, these cells were further purified using Lympholyte®-M. The activated DO11.10 lymphocytes were adoptively transferred to syngeneic BALB/c host mice. Uveitis was induced by intravitreal injection of cognate antigen OVA. Forty eight hours after the induction of uveitis in the recipient animals, the mice received an intraperitoneal injection of rhodamine to label circulating leukocytes, and uveitis was assessed as the infiltration of fluorescent leukocytes within the anterior uvea [22]. As shown in Figure 6A, intravitreal administration of OVA resulted in an influx of leukocytes in the eyes of BALB/c mice that received activated DO11.10 lymphocytes. No infiltrating inflammatory cells were observed in the eyes at 0 hour before OVA injection or in the control animals that received BSA (data not shown). Compared to the control DO11.10 cells, adoptive transfer of OX40 activating antibody-treated T cells significantly increased the number of both rolling and adhering leukocytes in the iris vasculature in response to OVA stimulation (Figure 6A), suggesting more ocular inflammation mediated by the T cells further activated by OX40 co-stimulation. However, roscovitine significantly inhibited OX40 activating antibody-augmented leukocyte rolling and adhesion in the adoptive transfer uveitis model (Figure 7A). 

OVA-induced uveitis elicits a rapid ocular immune response to a foreign antigen in the anterior chamber. To validate the effect of roscovitine on a self-antigen-mediated posterior uveitis, we used IRBP-induced EAU model. Compared to the control group, as we have previously reported [30], further activation of uveitogenic T cells by anti-OX40 activating antibody exacerbated ocular inflammation as evidenced by more severe EAU score (Figure 7B). Some OX40 activating antibody-treated B10.RIII mice also intraperitoneally received 10 μg roscovitine every other day from days 0 to 21 after IRBP immunization. Similar to the finding in OVA-induced uveitis, *in vivo* treatment of roscovitine mitigated EAU (Figure 6B). These data suggest that roscovitine inhibits pathological T lymphocytes even after their activation and function are enhanced by OX40 co-stimulation *in vivo*.

## Discussion

 CdKs are the key regulatory component of cell division. CdK4 and CdK6 control the G0 to mid G1 transition, whereas CdK2 facilitates the entry to S phase during division cycle. Thus, these CdKs could influence cell cycle progression during T cell expansion. In addition, recent studies have demonstrated that CdKs not only promote lymphocyte proliferation but also enhance T cell activation and differentiation. Certain CdKs such as CdK4 and CdK6 have been shown to play an important role in T cell differentiation [11,31], and several studies found that T cell activation depends on cell division [32-34]. Although the CdK2 endogenous inhibitor, p27(Kip1), is implicated in inducing T cell anergy and suppressing lymphocyte activation [12], the role of CdK2 in CD4+ T cell activation and function remains to be fully defined. In this study, our overall goal is to test targeting CdK2 as a novel strategy to modulate CD4+ T cell response and thus treat autoimmune uveitis. We confirmed the T cell activation by measuring the induction of OX40, a key co-stimulatory molecule involved in the enhancement of lymphocyte effector function and expansion. More activated CD4+ T cells entered into S phase, suggesting that they are in an active proliferation state. Coinciding with this observation, these proliferating lymphocytes expressed a higher level of CdK2. Moreover, roscovitine, a potent CdK2 inhibitor, blocked activation-induced T cell cycle progression. In addition, roscovitine reduced CD4+ lymphocyte activation and viability as evidenced by the data on OX40 and CD44 expression as well as annexin V/PI staining. These effects collectively contribute to the profound inhibition of inflammatory cytokine production by roscovitine as observed in this study. Lastly, we demonstrated the T cell suppressive effect of roscovitine using two T cell-dependent uveitis models. Treating mice with roscovitine significantly ameliorated ocular inflammation that was mediated by activated T cells. 

During T cell activation, co-stimulatory molecules provide a pivotal signal to the T cell response. OX40 is a crucial co-stimulatory molecule mainly expressed by activated T cells [1,2]. Using Scurfy mice that develop lethal autoimmune disease due to a profound defect of regulatory T cells, Lane et al. found that genetic deletion of CD30 and OX40 can reverse the fatal consequence caused by an unconstrained effector T cell response [35]. Knockout of CD30 alone had a very limited impact on the survival of Scurfy mice, suggesting that OX40 is primarily responsible for the activity of pathogenic T cells. Although lymphocytes express many other co-stimulatory molecules, this observation underscores the importance of OX40 in T cell activation and expansion. Recently, we have demonstrated a marked infiltration of OX40+ lymphocytes in human non-seeing eyes with chronic end stage uveitis [30]. To mirror the finding in patients, we also found an increase of OX40 transcription in antigen-induced mouse uveitis. In addition, activation of OX40 significantly augments uveitis, whereas blocking OX40 signaling substantially attenuates ocular inflammation [22]. OX40 promotes an autoimmune response via enhancing the effector function, expansion, and survival of pathogenic T cells. 

In this study, we used OX40 activating antibody to promote T cell activation and expansion. Our primary aim is to evaluate the suppressive effect of roscovitine on CD4+ cell division, viability, and activation. However, roscovitine appears to counteract the pro-activation and pro-proliferation actions of OX40 on T lymphocytes. This may explain our observation that roscovitine attenuated the ocular immune response mediated by OX40-activated T cells. As discussed previously, OX40 signaling can theoretically influence the expression and of cyclins and CdKs [4], and the integral role of CdK2 in T cell activation is in common with that of OX40. However, currently there is no direct evidence showing that OX40 signaling directly regulates CdK2 activity, and more research is needed to determine if CdK2 serves as an important intermediary to exert OX40 function. 

In this study, we used roscovitine to investigate the role of CdK2 in T cell activation. Although it is a potent CdK2 inhibitor by competing with the binding of ATP to the active site of CdK2, roscovitine also inhibits CdK5 [36,37]. However CdK2 and CdK5 control different phases of cell cycle progression. In fact, the role of CdK5 in cell cycle progression remains controversial. CdK5 has been shown to suppress cell cycle by forming a complex with a p27 protein [38]. In contrast, CdK2 controls the key step of S phase entry. Here, we showed that roscovitine inhibited G1 to S progression of activated T cells. In addition, our unpublished observations found no significant increase of CdK5 protein expression in the lymphocytes up to 72 hours after anti-CD3 and anti-CD28 antibody activation. Thus, suppressing CdK2 activity is likely responsible for the immune-inhibitory effect of roscovitine observed in this study. Nevertheless, future research using CdK2 and CdK5 KO models would help to more definitively characterize the target(s) of roscovitine in T cell-mediated pathology.

Lastly, we found that a higher concentration of roscovitine inhibited the expression of Gadd45β in activated lymphocytes. Since Gadd45β plays an important role in cell survival, suppression of Gadd45β can reduce the ability of T cells to endure stress, thereby enhancing apoptosis. In addition, recent studies have shown that T cells express Gadd45β upon the signaling of TCR or certain cytokines such as IL-12 and IL-18 [39]. Gadd45β is critical to maintain the homeostasis of the T cell response. Naïve T cells rapidly induce the expression of Gadd45β after activation. Gadd45β-deficient effector T cells exhibit an ablated cytokine production and cellular response to *in vitro* activation [40]. This suggests that Gadd45β is essential for the responsiveness and functionality of activated T cells. Here, we showed that roscovitine inhibited Gadd45β expression, T cell activation and cytokine production. This suggests that Gadd45β may potentially be a novel intracellular molecule targeted by roscovitine. We hope that this finding will promote more research to further elucidate the molecular mechanism by which roscovitine down-regulates T cell response.

In summary, we found that roscovitine induced cell cycle arrest and cell death of CD4+ lymphocytes. Moreover, it suppressed T cell activation as evidenced by the reduction of CD44 and OX40 expression as well as cytokine production. The combined effect is likely the mechanism by which roscovitine ameliorates the ocular T cell immune response. This study also suggests that inhibition of CdK2 and other potential CdKs is a promising strategy to treat uveitis and other T cell-mediated diseases. Roscovinte (Seliciclib) has entered a clinical trial (NCT00999401) to treat cancer patients due to its anti-proliferative effect. With its newly found immunomodulatory effect and tested safety profile, it is feasible to envision the application of roscovitine in treating certain autoimmune and inflammatory diseases.
