# Correction: Roscovitine Suppresses CD4+ T Cells and T Cell-Mediated Experimental Uveitis

**DOI:** 10.1371/annotation/a034fe32-c48c-4531-9e81-4947d5ac49d9

**Published:** 2013-12-03

**Authors:** Zili Zhang, Qi Liu, Konstantin S. Leskov, Xiumei Wu, Jie Duan, Gary L. Zhang, Mark Hall, James T. Rosenbaum

The recipient of the grant from the NIH was incorrectly omitted from the Funding Statement. The Funding Statement should read: "This research was supported by an NIH grant to Dr. Zili Zhang (R01 EY022937). The funders had no role in study design, data collection and analysis, decision to publish, or preparation of the manuscript." 

